# Supplementary figures and images for: Role and Modulation of TRPV1 in Mammalian Spermatozoa: An Updated Review
Source: Int J Mol Sci. 2021 Apr 21;22(9):4306. doi: 10.3390/ijms22094306 (PMC8122410; doi:10.3390/ijms22094306)

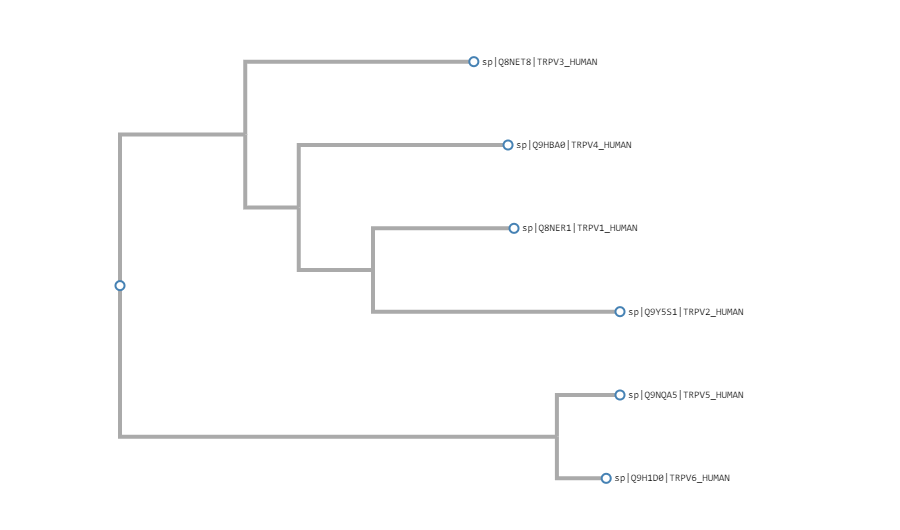

Supplement: Supplementary file 1 [file ijms-22-04306-s001.zip › ijms-1157372-supplementary.png]
